# A New Method of Predicting Final Mandibular Length Based on the Morphology of Cervical Vertebrae

**DOI:** 10.3390/diagnostics14242879

**Published:** 2024-12-21

**Authors:** Manami Yamaguchi, Yong-Il Kim, Heetae Park, Tetsutaro Yamaguchi

**Affiliations:** 1 Department of Orthodontics, School of Dentistry, Kanagawa Dental University, Yokosuka 238-8580, Japan; yamaguchi.manami@kdu.ac.jp (M.Y.); park@kdu.ac.jp (H.P.); t.yamaguchi@kdu.ac.jp (T.Y.); 2 Department of Orthodontics, School of Dentistry, Pusan National University, Yangsan 50612, Republic of Korea

**Keywords:** cephalogram, cervical vertebrae, diagnostic imaging, growth assessment, growth predictions, mandibular growth, mandibular length

## Abstract

Background/Objectives: Methods for predicting final mandibular length have been studied for many years. We aimed to estimate the final mandibular length at the end of the growth period by analyzing changes in cervical vertebral morphology using longitudinal lateral cephalograms. Methods: Longitudinal lateral cephalograms of elementary school students aged 6–15 who did not undergo orthodontic treatment, collected between 1965 and 1973, were used. For this analysis, 370 images from 44 female individuals were selected, and cervical vertebral morphology was assessed using the semi-landmark method. Generalized Procrustes analysis and principal component analysis were performed, and changes in maxillofacial skeletal and cervical vertebral morphology were analyzed using a linear mixed model with repeated measures. A predictive formula for estimating final mandibular length was developed based on morphological changes in the fourth cervical vertebra. Results: The difference between the predicted and actual final mandibular lengths using the semi-landmark method was 0.17 ± 0.08 mm. The marginal R^2^ value of the prediction formula was 0.957, and the conditional R^2^ was 0.990, demonstrating very high accuracy. The annual increase in mandibular length remained consistent each year but slowed after 14 years of age. Geometric morphometric analysis revealed significant morphological changes in the fourth cervical vertebra at 14 years of age, coinciding with a deceleration in mandibular growth. Conclusions: A highly accurate formula was developed to predict final mandibular length based on morphological changes in the fourth cervical vertebra. Cervical vertebral morphology may provide valuable information related to mandibular growth during adolescence.

## 1. Introduction

In orthodontic treatment, accurate assessment and prediction of maxillofacial skeletal growth and development are crucial for establishing proper diagnoses, setting treatment goals, and determining appropriate treatment options. These factors significantly impact the effectiveness of treatment and outcome [1,2,3]. Consequently, predicting the final morphology of the maxillofacial skeleton has long been a subject of debate and research [4,5].

Various approaches using lateral cephalograms have been explored, including partial least squares [4,6], growth potential method [7], growth curve method [7], multivariate analysis [7], Bayesian theorems [5], and machine learning-based prediction models [8,9]. However, accurately predicting individual growth-related changes in the maxillofacial skeleton remains complex and challenging [5]. A more reliable method is needed to predict maxillofacial growth and development without exposing patients to additional radiation [3].

Cervical vertebrae, visible in routinely used lateral cephalograms, offer a potential solution to this challenge [10,11]. Their proximity to the mandible and the similar development timing suggest a close relationship between these structures [12].

Sato et al. [7] compared six methods for predicting final mandibular length from cervical vertebrae, including (1) growth rate method using bone maturity of the hand bones based on the computer-assisted skeletal maturity assessment system they developed; (2) growth rate method using bone maturity of the hand bones based on the Tanner–Whitehouse 2 method; (3) growth potential method using bone maturity of the hand bones based on the Tanner–Whitehouse 2 method; and (4) growth rate method using growth curves. Their findings indicated that the growth potential method provided the most accurate predictions. This method predicts the final mandibular length by estimating the increment from the current mandibular length to the final length, necessitating a step to predict this increment.

Later, Mito et al. [3] improved this method by assessing the maturity of the cervical vertebrae [13]. They used a regression equation based on height and length measurement ratios and age. However, bone maturity evaluations, even by the same observer, can vary [14,15], making it difficult to reduce variability in growth prediction of the maxillofacial skeleton during the growth period. Therefore, assessing detailed changes and individual differences in cervical vertebral morphology due to growth remains challenging, and these variations may not be fully reflected in the predictions.

In this study, we used geometric morphometrics to directly evaluate and analyze changes in cervical vertebral morphology, enabling us to predict the final mandibular length.

Shapes derived from semi-landmarks were superimposed through generalized Procrustes analysis, and their variations were analyzed via principal component analysis (PCA). The objective was to estimate the final mandibular length (Condyle–Gnathion) at the end of the growth period based on cervical vertebral morphology changes observed in longitudinal lateral cephalograms of individuals who had not undergone orthodontic treatment.

## 2. Materials and Methods

### 2.1. Data Source

This study utilized longitudinal data on dent maxillofacial growth and development from the Department of Orthodontics, School of Dentistry, Kanagawa Dental University, Japan. The dataset comprised annual lateral cephalograms of elementary school students who did not undergo orthodontic treatment, aged 6–15 years (9.9 ± 2.7 years), collected between 1965 and 1973. For this analysis, 370 images from 44 female individuals were selected. Consent was obtained from the participants via an opt-out process. This study adhered to the Declaration of Helsinki guidelines and was approved by the Kanagawa Dental University Research Ethics Review Committee (approval number: 892; date of approval: 23 January 2023).

### 2.2. Cephalometric Measurements

**Figure 1:**
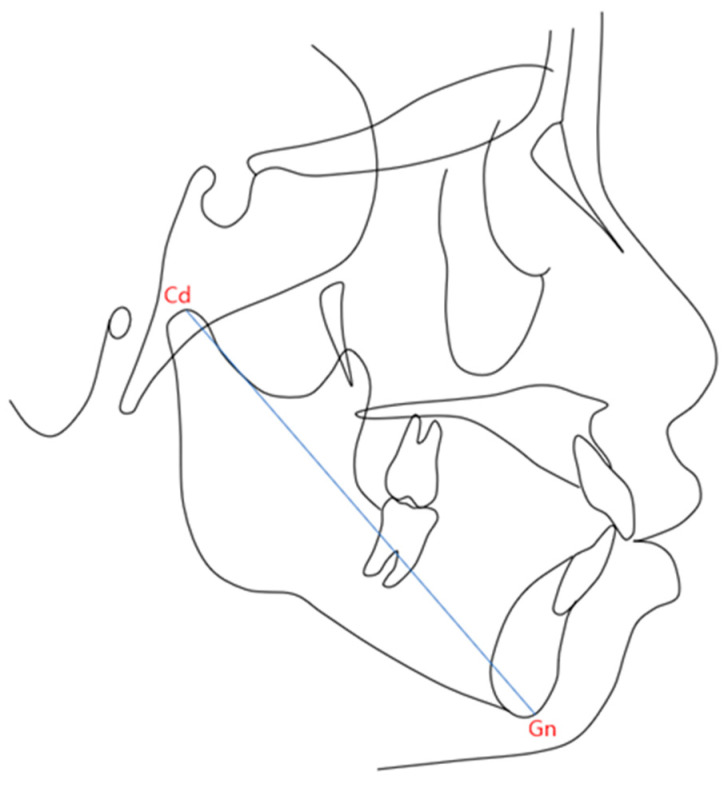
Cephalometric landmarks and linear measurements: Cd: This point represents the most superior point on the mandibular condyle; Gn: This point indicates the most anterior and inferior point on the mandibular contour; Cd-Gn: The blue line represents the distance between the Cd and the Gn, which is defined as the mandibular length.

For consistency, all measurements were conducted by a single examiner. The mandibular length was measured on digital cephalograms using CephaloMetrics AtoZ ver. 20.00 software (YASUNAGA Computer Systems Co., Inc., Fukui City, Fukui, Japan) without magnification. The following landmarks were defined:

- Condyle (Cd): The most superior point on the mandibular condyle.
- Gnathion (Gn): The most anterior and inferior point of the mandibular contour, determined by bisecting the angle formed by the mandibular border (Menton-Gonion) and the facial plane (Nasion–Pogonion). The distance between Cd and Gn was defined as the mandibular length (Figure 1).

### 2.3. Cervical Vertebrae Analysis

**Figure 2:**
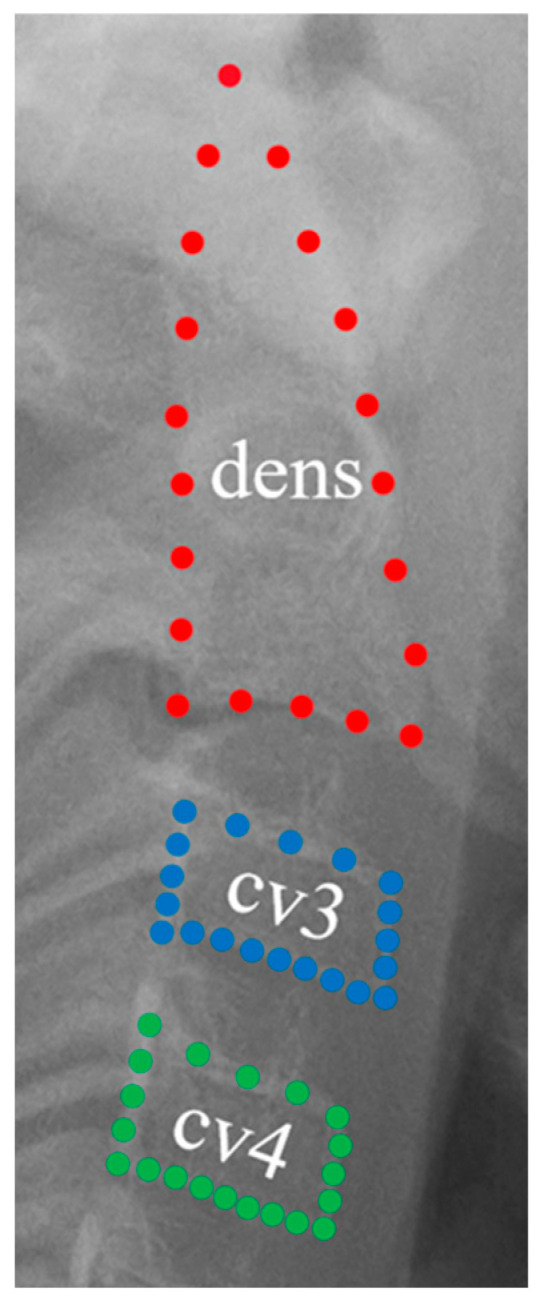
Cervical landmarks: Red points; these 20 landmarks correspond to the odontoid process (dens) of the second cervical vertebra; Blue points: these 20 landmarks represent the vertebral body of the third cervical vertebra (cv3); Green points: these 20 landmarks indicate the vertebral body of the fourth cervical vertebra (cv4).

The shapes of the second, third, and fourth cervical vertebrae (cv) were measured using the semi-landmark method. In total, 60 landmarks were obtained. The second cervical vertebra’s odontoid process had 20 landmarks, while the third and fourth vertebral bodies had 20 each. Landmark coordinates were extracted using ImageJ software V. 1.53t (National Institutes of Health, Bethesda, MD, USA) (Figure 2). Morphological changes in the cervical vertebrae were evaluated based on landmark coordinates rather than linear measurements or sizes.

**Figure 3:**
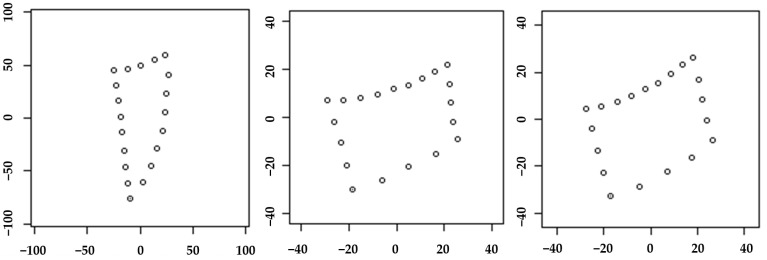
Generalized Procrustes analysis and PCA. From the left, these are the dens, the third cervical vertebra, and the fourth cervical vertebra.

Generalized Procrustes analysis and PCA were performed. This geometric morphometric approach utilized semi-landmark coordinates as raw data. Procrustes’ analysis eliminated the effects of rotation and translation through superimposition (Figure 3). Subsequently, mandibular length was predicted based on Cd-Gn measurements and PCA results for each cervical vertebra.

### 2.4. Statistical Analysis

All coordinates were utilized for geometric morphometric analysis, and linear mixed models were developed to predict mandibular length using Cd-Gn measurements and PCA results for each cervical vertebra. In this model, the mandibular length (Cd-Gn) served as the dependent variable, while age and principal component (PC) scores representing the shapes of the second, third, and fourth cervical vertebrae were incorporated as predictor variables. This historical dataset (1965–1973) of longitudinal cephalograms from untreated subjects is unique and valuable for research. Current ethical guidelines restrict collecting similar longitudinal radiographic data from untreated control groups due to concerns about radiation exposure. This makes our dataset particularly significant for understanding natural growth patterns, though we acknowledge its temporal limitations.

## 3. Results

To assess measurement reliability, Cd-Gn measurements were re-measured by the same researcher after a two-week interval on 30 randomly selected cephalograms [16]. Measurement error was calculated using Dahlberg’s formula [17].

The analysis yielded an Intraclass Correlation Coefficient of 0.99 with a random error of 0.19 mm for Cd-Gn measurement, indicating exceptionally high intra-observer reproducibility of the Cd-Gn measurements. All statistical analyses were conducted using R Studio: Integrated Development for R (RStudio, PBC, Boston, MA, USA). The following R packages were utilized: lme4, lmerTest, and sjPlot.

**Figure 4:**
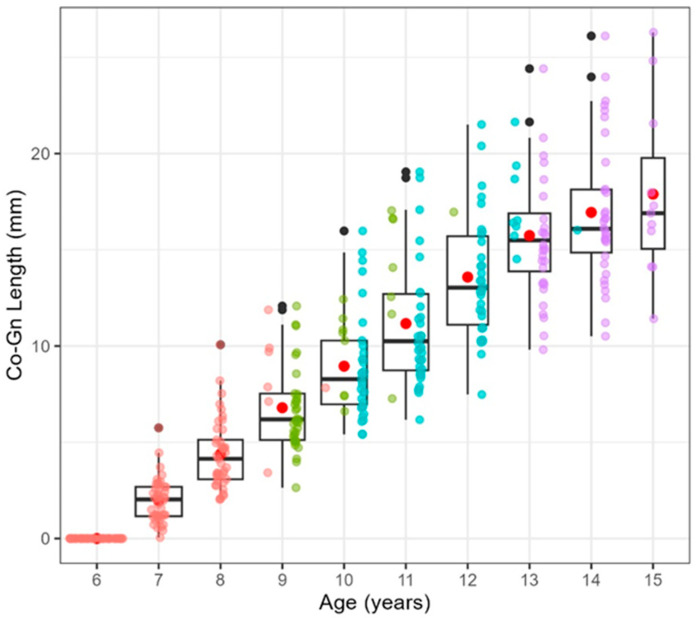
Spearman’s rank correlation coefficient between age and mandibular length.

A Spearman’s correlation coefficient of 0.932 was identified between mandibular length and chronological age among female participants (Figure 4), denoting a robust positive correlation. From these results, it can be confirmed that the tendency for the mandible to grow becomes stronger with age, and even after considering growth stages and individual differences, the growth pattern is highly stable.

### 3.1. Statistical Shape Analysis

Using generalized Procrustes analysis, we extracted and compared the mean morphologies of the second, third, and fourth cervical vertebrae, including the odontoid process of the second cervical vertebra and the vertebral bodies of the third and fourth cervical vertebrae, based on their geometric shape data.

### 3.2. Linear Mixed Model for Mandibular Length Prediction

In this study, a mixed-effects model was employed to explore the relationship between the dependent variable (mandibular length) and key predictors, incorporating both fixed and random effects. The fixed effects included age adjusted by 6 years (age-6), the initial value adjusted by its mean (initial value adjusted mean), an interaction term between age-6 and the adjusted initial value, and a PC derived from a covariate, specifically cv4 PC1.

Principal Component (PC) scores represent the key patterns of shape variation in the cervical vertebrae after dimensional reduction through Principal Component Analysis (PCA). In our study, each PC captures a specific aspect of morphological variation: PC1 accounts for the largest percentage of shape variation, primarily representing changes in vertebral body height and width ratios. These scores quantify how much each individual vertebra deviates from the mean shape along the primary axis of variation. Let me clarify: In our initial analysis, we examined all three vertebrae (cv2, cv3, and cv4) using geometric morphometrics. However, our statistical analysis revealed that cv4 showed the strongest correlation with mandibular growth patterns and provided the most reliable predictive value. This led us to focus on cv4 for the final predictive model. Let me clarify: In our initial analysis, we examined all three vertebrae (cv2, cv3, and cv4) using geometric morphometrics. However, our statistical analysis revealed that cv4 showed the strongest correlation with mandibular growth patterns and provided the most reliable predictive value. This led us to focus on cv4 for the final predictive model. For the predictive model, we used PC scores from cv4 as they provided quantitative measures of shape variation that correlated strongly with mandibular growth patterns.

The model intercept was estimated at 82.850 (95% confidence interval [CI]: 82.58–83.11, *p* < 0.001), indicating that when all other predictors are held constant, the expected mandibular length is 82.850. This intercept was statistically significant (*p* < 0.001).

The age-6 variable had a positive association with mandibular length, with an estimate of 2.250 (95% CI: 2.12–2.37, *p* < 0.001), indicating that for each additional year beyond 6 years of age, the mandibular length increased by an average of 2.250 units. This effect was highly significant (*p* < 0.001).

The initial_value_adj_mean variable, which represents the standardized mean mandible length during early growth stages, accounting for interindividual variability, significantly impacted subsequent growth. With an estimate of 1.010 (95% CI: 0.98–1.04, *p* < 0.001), this finding indicates that for every unit increase in the adjusted initial value, the mandibular length increased by approximately 1.010 units, controlling for other variables. The narrow CI and significant *p*-value observed highlight the robustness of this association.

The interaction term between age-6 and the adjusted initial value was also included in the model, with an estimated coefficient of 0.040 (95% CI: 0.02–0.05, *p* < 0.001). This positive interaction suggests that the effect of age on mandibular length is amplified by the initial value, with higher initial values leading to a more pronounced age-related increase in mandibular length.

The interaction term (visit × initial value adj) further illustrates the combined influence of age and initial value on mandibular growth. Specifically, a larger initial value accelerates growth as age increases. The cv4 PC1, derived from PCA, indicated morphological differences and showed a negative association with mandibular growth, suggesting a slight inhibitory influence. Overall, the initial values adjusted for age-6 and mean had a significant positive impact on mandibular length, with high statistical robustness (Table 1).

Predictive mandibular length formula = 82.849 + 2.247 × (age-6) + 1.015 × initial_value_adj_mean + −0.324 × cv4.PC1_ + 0.039 × (age-6)(1)

The initial values adjusted for age-6 and mean exhibited a positive and highly significant effect on the dependent variable. The model also included an interaction term between age-6 and the adjusted initial values, and this positive interaction suggests the impact of age on the dependent variable is moderated by the initial values. Specifically, the interaction suggests that as initial values increase, the influence of age on mandibular growth becomes more pronounced.

Conversely, cv4 PC1 showed a statistically significant negative association with the outcome, where for every 1-unit increase in this PC, the dependent variable decreased by 0.320 units. The negative coefficient of cv4 PC1 (−0.320) indicates that certain vertebral shape characteristics are associated with reduced mandibular length. The cv4 PC1 captures shape variability through PCA, and its inverse relationship with mandibular growth implies that it reflects a morphological dimension that constrains mandibular development.

The model also included random effects to account for variability across different levels of the data, enhancing its robustness. The explanation power of the model was assessed using marginal and conditional R^2^ values, which demonstrated that a substantial portion of the variance in the dependent variable was explained by the predictors. Model fit was further evaluated using statistical criteria, such as deviance, Akaike Information Criterion (AIC), its corrected version (AICc), log-likelihood, and Bayesian Information Criterion (BIC). Lower AIC and BIC values indicated that the model fit the data well, showing better performance.

**Figure 5:**
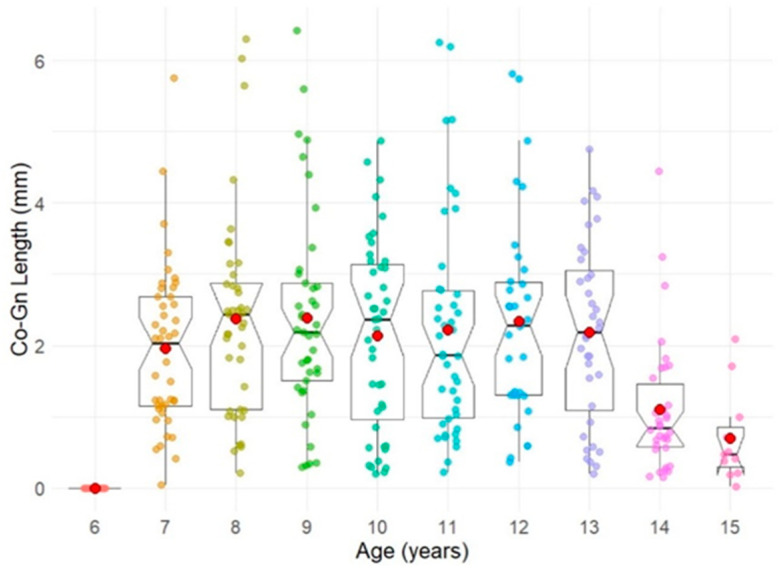
Change in mandibular growth.

Moreover, a decline in mandibular growth increment was observed around the age of 14 years (Figure 5), which aligns with the expected reduction in mandibular growth as maturity is reached. This figure shows the annual increase in mandibular length by age, using the age of 6 years as the baseline. As age increases, there is a general tendency for the annual increase in mandibular length to decrease. This trend is particularly pronounced between the ages of 14 and 15 years.

## 4. Discussion

This study demonstrated a novel method for predicting final mandibular length using cervical vertebral morphology analysis [2]. Lateral cephalograms, routinely used in orthodontics, allow for the tracing of these structures [18], forming the basis for diagnosis and treatment planning. Studying the changes in cervical vertebral morphology due to growth is beneficial for orthodontists in improving treatment outcomes [3].

In this study, we employed the semi-landmark method to measure cervical vertebral morphology, eliminating additional radiation exposure. After performing generalized Procrustes and PCA, changes were analyzed using a linear mixed model to predict the final mandibular length. The developed predictive model, based on a fourth cervical vertebra (cv4) morphological changes, achieved high accuracy with a mean prediction error of 0.17 ± 0.08 mm (marginal R^2^ = 0.957, conditional R^2^ = 0.990). Our analysis revealed significant morphological changes in cv4 at age 14, corresponding with mandibular growth deceleration. The semi-landmark method provided more precise measurements compared to traditional approaches, reducing measurement variability. The predictive formula incorporating cv4 morphological changes offers clinicians an objective tool for estimating the final mandibular length without additional radiation exposure. While our findings are based on female Japanese subjects from historical data, this methodology establishes a foundation for future studies across different populations and genders. The key factor contributing to this enhanced accuracy was attributed to the use of the semi-landmark method for cervical vertebrae morphometrics. This method allows for precise capture of subtle vertebral shape differences, reducing measurement variability [19]. By minimizing measurement error and capturing fine morphological changes, a more sensitive and accurate predictive model was developed.

A key feature of this study is the longitudinal observation of growth in Japanese individuals who have not undergone orthodontic treatment [20]. As orthodontic treatment can potentially alter natural maxilla and mandible growth patterns, studying untreated individuals is crucial. However, due to ethical constraints, obtaining lateral cephalograms from untreated patients for research is challenging, making this dataset particularly valuable. Furthermore, by focusing on Japanese subjects, the findings are directly applicable to clinical practice in Japan, and this approach also lays the groundwork for future evaluations comparing growth pattern differences across different ethnic groups [21,22,23].

In this study, we compared morphological changes in the odontoid process, the third cervical vertebra (cv3), and the fourth cervical vertebra (cv4) to predict the final mandibular length. The results revealed that cv4 demonstrated the highest predictive accuracy. Growth-related morphological changes occur in all vertebrae from the atlas (cv1) to the seventh cervical vertebra (cv7) [24,25,26]; however, the degree and nature of these changes vary among the vertebrae. For example, the odontoid process shows minimal change [24,27], while cv3 and cv4 initially have similar trapezoidal shapes [25,26]. However, cv4 showed more pronounced changes in the height of the anterior and posterior surfaces of the vertebral body compared to cv3 [28]. This observation, supported by principal component analysis, suggests that the morphological changes associated with cv4 are more significant than those of other cervical vertebrae during the growth period.

This study provides valuable insights into maxillofacial growth patterns, particularly focusing on the period between 6 and 15 years of age [29,30]. This study provides insights into female growth patterns during the age range of 6–15 years, which encompasses significant developmental changes. Future research including male subjects would be valuable for gender-based comparisons of growth patterns. This finding is particularly relevant given that the studied age range (6–15 years) encompasses a critical period of significant growth in female individuals, a factor carefully considered in our evaluation. A key strength of our methodology was the use of longitudinal lateral cephalograms from untreated individuals, which enabled us to accurately track individual growth trajectories over time. By employing the semi-landmark method to measure cervical vertebrae, we were able to quantitatively assess changes in cervical vertebral morphology through coordinate changes, ultimately deriving a predictive equation for final mandibular length based solely on objective data.

Our analysis of the fourth cervical vertebra (cv4) revealed a significant finding: we identified periods when morphological changes in cv4 were most pronounced. This discovery presents a potential method for predicting the timing of mandibular growth changes, which could have substantial implications for orthodontic treatment planning. Such insights could allow for more precise timing of orthodontic interventions, aligning them more closely with an individual’s unique growth patterns.

An important consideration for interpreting our results is the historical nature of our dataset (1965–1973). Secular trends in growth and development over the past 60 years may affect the direct application of our findings to contemporary populations. Environmental factors, nutritional changes, and general health improvements have influenced growth patterns globally. Japanese populations, in particular, have shown significant secular trends in height and development timing during the post-war period through the early 21st century.

While our methodology for analyzing cervical vertebral morphology remains valid, clinicians should consider potential secular changes when applying our specific measurements to modern patients. Future studies using contemporary datasets would be valuable for validating and potentially adjusting our predictive model for current populations.

While this study provides valuable insights into craniofacial growth patterns, it is essential to acknowledge its limitations and identify avenues for future research. The study’s primary limitation lies in its data source: the findings are based exclusively on a small historical Japanese female sample aged 6–15 years. Therefore, inferences drawn from these results must be interpreted with caution when applying them to contemporary general populations. The study’s focus on a specific age group (6–15 years) and its inclusion of exclusively female participants yielded important findings. However, to enhance the clinical applicability of these results, future research should address several key areas. Including male participants in similar longitudinal studies would provide a more comprehensive understanding of growth patterns across genders. Given the known differences in growth trajectories between male and female individuals, elucidating these distinctions within the Japanese population would offer valuable insights for clinical practice, enabling more tailored orthodontic approaches based on gender-specific growth trends.

Additionally, conducting comparative studies across different racial and ethnic groups could reveal potential variations in growth patterns, further enhancing our understanding of craniofacial development. Such research would pave the way for more personalized orthodontic treatment approaches for diverse patient populations.

## 5. Conclusions

This study provides a foundation for predicting final mandibular length using objective measurements of cervical vertebral morphology, with a particular focus on cv4. As we continue to refine and expand upon these methods, we anticipate that this line of research will contribute to more precise and personalized orthodontic treatment strategies in the future, ultimately improving patient outcomes across diverse populations.

## Figures and Tables

**Table 1:** Predictive model for mandibular length using linear mixed model.

Predictors (n = 370)	Estimates	Confidence Interval	*p*-Value
(Intercept)	82.850	82.58–83.11	<0.001
age-6	2.250	2.12–2.37	<0.001
initial value adjust mean	1.010	0.98–1.04	<0.001
(age-6) × initial value adj	0.040	0.02–0.05	<0.001
cv4 PC1	−0.320	−0.47–−0.18	<0.001
**Random Effects**			
σ^2^	1.2		
τ_00_	0.19_id_		
τ_11_	0.14_id.visit_		
ρ_01_	0.55_id_		
N	44_id_		
Marginal R^2^/Conditional R^2^	0.957/0.990		
Deviance	1263.838		
AIC	1305.775		
AICc	1306.275		
log-Likelihood	−643.887		
BIC	1328.435		

Intercept: This represents the estimated mandible length when all explanatory variables are held constant, effectively excluding the influence of factors like age or initial values. Visit (Age): This variable is critical, indicating that age significantly impacts mandibular length. σ^2^: This term reflects the residual variance, quantifying the level of observation error within the random effects model or the variability not explained by the model. τ_0__0_ (id): This represents the random effects variance at the individual level, indicating the extent of baseline differences between individuals. τ_1__1_ (id.visit): This denotes the random effects variance across both individuals and visits, indicating the magnitude of variation attributable to differences in visits within individuals. ρ_0__1_ (id): This coefficient represents the correlation between random effects at the individual level, specifically between τ_0__0_ and τ_1__1_. For this study, a value of 0.55 suggests a moderately positive correlation. N (id): This denotes the sample size, with 44 individuals included in the model.

## Data Availability

The data presented in this study are available from the corresponding author upon request.
